# Rapid enlargement of a tubo-ovarian abscess in a patient with cervical cancer and multiple uterine fibroids: A case report

**DOI:** 10.1016/j.crwh.2026.e00784

**Published:** 2026-01-06

**Authors:** Yuki Yamazawa, Takayoshi Iijima, Daisuke Shigenaga, Tamaki Cho, Yuichi Imai, Taichi Mizushima, Etsuko Miyagi

**Affiliations:** a Department of Obstetrics and Gynecology, Yokohama City University Hospital, Yokohama, Japan; b Department of Radiation Oncology, Yokohama City University Hospital, Yokohama, Japan

**Keywords:** Tubo-ovarian abscess, Cervical cancer, Uterine fibroids, Concurrent chemoradiotherapy

## Abstract

Locally advanced cervical cancer can be complicated by infections such as pyometra and tubo-ovarian abscess (TOA), although the pathogenesis of TOA is not fully understood. This report presents a case of TOA that rapidly enlarged in a patient with cervical cancer and multiple intramural uterine fibroids. A 61-year-old primigravida was diagnosed with stage IIB cervical cancer, classified as cT2bN0M0, squamous cell carcinoma, complicated by multiple fibroids. Concurrent chemoradiotherapy (CCRT) was planned, and the patient was admitted three weeks after her initial visit with lower abdominal pain. Computed tomography revealed an 8 cm TOA, pyometra, and an enlarged appendix. Conservative treatment with trans-cervical drainage and intravenous antibiotics was unsuccessful. Surgical drainage with bilateral adnexectomy and appendectomy was therefore performed. *Bacteroides fragilis* was identified in intra-abdominal pus cultures, consistent with findings from cervical drainage, suggesting that the TOA developed secondary to pyometra. One month after surgery, CCRT was resumed and completed without complications. TOA associated with cervical cancer can enlarge rapidly and may require surgical drainage. In patients with locally advanced cervical cancer and multiple uterine fibroids, the potentially increased risk of TOA should be considered.

## Highlights

• Tubo-ovarian abscess (TOA) is a rare complication of cervical cancer.
• The presence of multiple uterine fibroids may contribute to rapid progression of TOA in patients with cervical cancer.
• If conservative treatment for TOA fails, prompt surgical drainage is required to avoid delaying cervical cancer treatment.

## 1 Introduction

Cervical cancer has the fourth highest incidence and mortality rates of all cancers among women worldwide [1]. In 2022, more than 660,000 women were diagnosed with cervical cancer, and over 480,000 died from the disease. Locally advanced cervical cancer may be complicated by infections such as pyometra and tubo-ovarian abscess (TOA). The incidence and natural history of TOA in patients with cervical cancer remain unclear. Only a few case reports have been published, including one describing two cases of cervical cancer with TOA among 93 total cases [2].

Treatment options for TOA include conservative management with antibiotics, surgical drainage, and CT-guided aspiration. Risk factors for failure of conservative treatment include older age, postmenopausal status, and large abscess size [3]. In the present case, a TOA enlarged rapidly during the short interval between diagnosis and the initiation of treatment. Multiple uterine fibroids were suspected to have contributed to this rapid progression.

## 2 Case Presentation

**Fig. 1:**
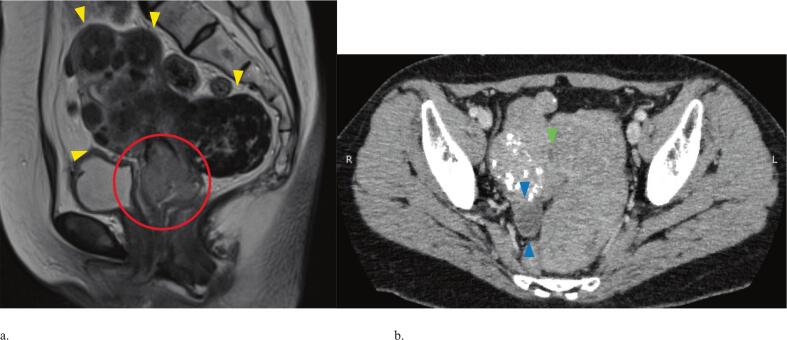
a. Pelvic magnetic resonance image at first visit showed multiple uterine fibroids, with the largest 8 cm in diameter (yellow arrowheads), and a cervical mass with parametrial infiltration (red circle). b. Computed tomography at first visit showed a 2 cm right hematosalpinx (blue arrowheads), no pyometra (green arrowhead). (For interpretation of the references to colour in this figure legend, the reader is referred to the web version of this article.)

A 61-year-old primigravida presented with abnormal bleeding and was referred to hospital. A 4 cm cervical mass was identified, and biopsy confirmed squamous cell carcinoma. CT imaging revealed a 2 cm right hematosalpinx without lymphadenopathy or distant metastasis. MRI demonstrated multiple intramural uterine fibroids, the largest 8 cm in diameter, along with a cervical mass showing parametrial infiltration. Based on these findings, the diagnosis was stage IIB cervical cancer (International Federation of Gynecology and Obstetrics (FIGO) 2018), classified as cT2bN0M0 (Fig. 1 a, b). Initial blood test results were: CRP 0.02 mg/dL, white blood cell count 5700/μL, and SCC 3.0 ng/mL. The planned treatment was concurrent chemoradiotherapy (CCRT).

**Fig. 2:**
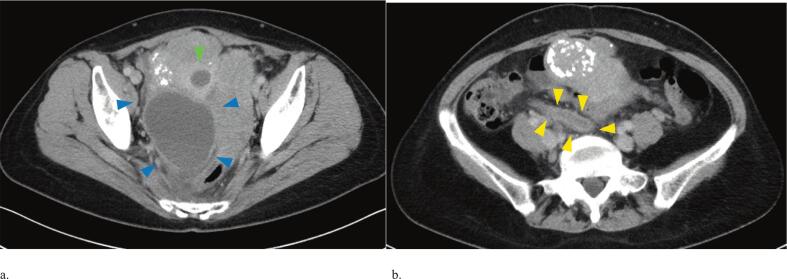
a. Computed tomography at admission showed an 8 cm right TOA (blue arrowheads), pyometra (green arrowhead). b. Computed tomography showed an enlarged appendix (yellow arrowhead). (For interpretation of the references to colour in this figure legend, the reader is referred to the web version of this article.)

The patient was admitted three weeks after her initial visit. On admission, she complained of lower abdominal pain. Blood tests showed marked inflammation with CRP 21.54 mg/dL and white blood cell count 15,500/μL. CT revealed a right-sided 8 cm TOA, pyometra, an enlarged appendix, and ascites (Fig. 2 a, b). Whole-pelvis irradiation was started at 1.8 Gy per day on hospital days 1 and 2 (total 3.6 Gy). Treatment was then discontinued after TOA was diagnosed. Conservative therapy with trans-cervical drainage and intravenous antibiotics was initiated. *Bacteroides fragilis* was cultured from pus obtained by cervical drainage.

**Fig. 3:**
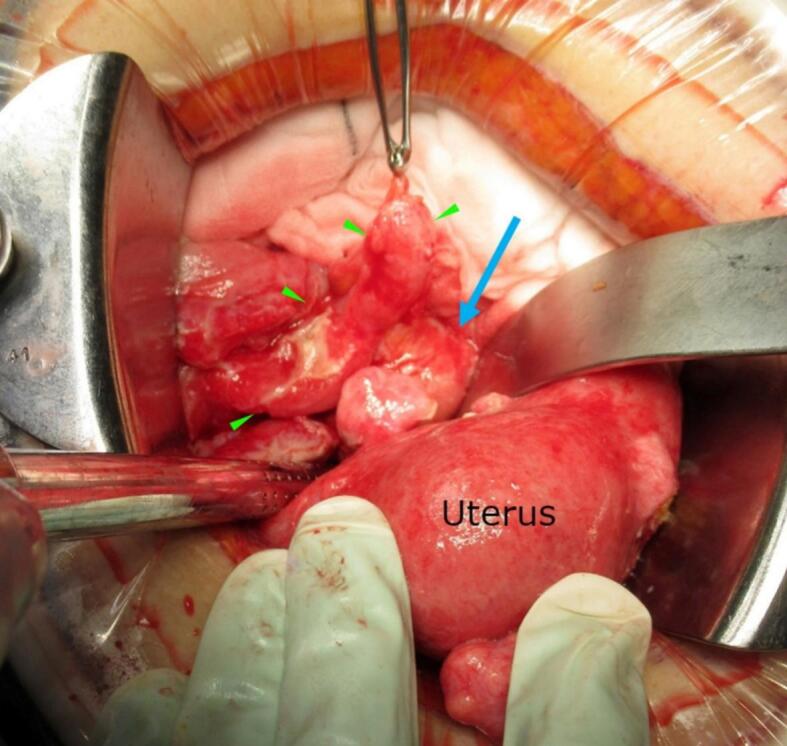
Macroscopic appearance of right TOA. The 8 cm TOA (blue arrow) was highly adherent to the surrounding peritoneum and enlarged appendix (green arrowheads). (For interpretation of the references to colour in this figure legend, the reader is referred to the web version of this article.)

Because her symptoms did not improve, surgical drainage was scheduled on hospital day 4. At laparotomy, the right fallopian tube was markedly enlarged and adherent to an enlarged appendix and the right adnexa. A large amount of purulent fluid was present, but the uterus was intact without rupture. Bilateral adnexectomy, appendectomy, and copious irrigation of the peritoneal cavity with saline were performed (Fig. 3).

Pathology of the right adnexa showed neutrophil infiltration and abscess formation. The appendix displayed neutrophil infiltration extending to the serosal layer with intact mucosa, indicating spread of inflammation from the TOA. Cultures from intra-abdominal pus yielded *Bacteroides fragilis*, consistent with the organism identified in the cervical drainage.

One month after surgery, CCRT was resumed. The patient received whole-pelvis three-dimensional conformal radiotherapy with 41.4 Gy in 23 fractions, followed by central shielding (a midline block technique commonly used in Japan) with 9 Gy in 5 fractions, and intracavitary brachytherapy with 24 Gy in 4 fractions (point A prescription). Although the post-appendectomy ileocecal region was within the irradiation field, treatment was completed without complications. At one year after completion of therapy, the patient remained in complete remission under follow-up.

## 3 Discussion

TOA associated with cervical cancer and multiple uterine fibroids can enlarge rapidly. If conservative treatment fails, surgical drainage should be performed promptly to avoid delaying cervical cancer therapy.

In this case, multiple uterine fibroids may have contributed to the rapid progression of TOA. Cervical cancer is often complicated by pyometra caused by obstruction of vaginal discharge due to a cervical mass; however, only a few reports have described cases associated with TOA. Among 61 postmenopausal women with TOA, 8 (13.1 %) had malignant tumors, but no cases of cervical cancer were involved [4]. Barton et al. reported three cases of TOA complicating cervical cancer [5]. All three patients presented with symptoms such as fever and lower abdominal pain at their initial visit, suggesting a pre-existing infection; therefore, the pathogenesis and course of abscess progression remain unclear.

Uterine fibroids may have influenced the clinical course in this case, and there is a report that 13 % of postmenopausal women with abscesses have uterine fibroids larger than 5 cm [2].

In the present case, multiple intramural fibroids extended from the uterine fundus to the uterine body, and both pyometra and TOA were present. Multiple intramural uterine fibroids likely hindered uterine distension, resulting in extension of the intrauterine abscess into the fallopian tube, with subsequent spread of pus into the peritoneal cavity. This is supported by the intra-abdominal findings, which revealed no rupture of the uterus, and by the identical culture results from cervical drainage and intra-abdominal pus collected during surgery. The TOA progressed rapidly within only three weeks from the initial consultation.

Management of TOA includes both conservative and surgical approaches. Surgical treatment should not be delayed when conservative management proves ineffective. Risk factors for failure of conservative treatment include an abscess diameter of 5 cm or greater and postmenopausal status [6,7]. In the present case, conservative therapy was initiated to avoid delaying cervical cancer treatment; however, surgical drainage was ultimately required. Given that the abscess measured 8 cm at the start of treatment, primary surgical intervention may have been more appropriate. In addition, although appendiceal enlargement was observed on CT in the present case, intraoperative findings were reviewed in consultation with a gastrointestinal surgeon and were considered to reflect secondary inflammatory spread from the TOA. Pathological examination also supported inflammatory extension originating from the TOA. Multidisciplinary collaboration should be considered during surgical management. Less invasive alternatives to open surgical drainage include interventional radiology (IR) drainage and laparoscopic surgery. In a reported series of 19 postmenopausal women with TOA who underwent IR drainage, 9 cases (47.4 %) eventually required open surgical drainage [4]. In the present case, the presence of acute peritonitis and the need for thorough intraperitoneal lavage led to the choice of open surgery.

Although delayed wound healing is a known complication of radiation therapy, it is unclear whether CCRT following intestinal surgery increases the risk of anastomotic failure. A prospective cohort study of postoperative radiation therapy for rectal cancer showed an anastomotic leakage rate of 17.0 % in patients who received postoperative radiation and 11.5 % in those who did not (*P* = 0.245), indicating that postoperative radiation therapy was not a risk factor for leakage [8]. The optimal interval between surgery and radiotherapy has not been sufficiently studied. In this case, CCRT was resumed four weeks after bilateral adnexectomy and appendectomy, and treatment was completed without complications.

## 4 Conclusion

TOA associated with cervical cancer and multiple fibroids can enlarge rapidly and require heightened clinical vigilance. In patients with locally advanced cervical cancer and coexisting multiple uterine fibroids, the increased risk of TOA should be considered. When conservative treatment is ineffective, prompt surgical intervention should be undertaken to avoid delaying definitive cervical cancer therapy and to improve clinical outcomes.

## Contributors

Yuki Yamazawa contributed to patient care, acquiring and interpreting the data, drafting the manuscript, and undertaking the literature review.

Takayoshi Iijima contributed to patient care, conception of the case report, drafting the manuscript, and revising the article critically for important intellectual content.

Daisuke Shigenaga contributed patient care and revising the article critically for important intellectual content.

Tamaki Cho contributed to patient care and revising the article critically for important intellectual content.

Yuichi Imai contributed to patient care and revising the article critically for important intellectual content.

Taichi Mizushima contributed to revising the article critically for important intellectual content.

Etsuko Miyagi contributed to revising the article critically for important intellectual content.

All authors approved the final submitted manuscript.

## Patient consent

Written informed consent was obtained from the patient for the publication of the case report and accompanying images.

## Provenance and peer review

This article was not commissioned and externally peer reviewed.

## Funding

No funding from an external source supported the publication of this case report.

## Declaration of competing interest

The authors declare that they have no competing interest regarding the publication of this case report.
